# Descriptive Comparison of ELISAs for the Detection of *Toxoplasma* *gondii* Antibodies in Animals: A Systematic Review

**DOI:** 10.3390/pathogens10050605

**Published:** 2021-05-15

**Authors:** K. L. D. Tharaka D. Liyanage, Anke Wiethoelter, Jasmin Hufschmid, Abdul Jabbar

**Affiliations:** Department of Veterinary Biosciences, Melbourne Veterinary School, Faculty of Veterinary and Agricultural Sciences, The University of Melbourne, Werribee, VIC 3030, Australia; anke.wiethoelter@unimelb.edu.au (A.W.); huj@unimelb.edu.au (J.H.); jabbara@unimelb.edu.au (A.J.)

**Keywords:** toxoplasmosis, animals, ELISA, native antigens, recombinant antigens

## Abstract

*Toxoplasma gondii* is the zoonotic parasite responsible for toxoplasmosis in warm-blooded vertebrates. This systematic review compares and evaluates the available knowledge on enzyme-linked immunosorbent assays (ELISAs), their components, and performance in detecting *T. gondii* antibodies in animals. Four databases were searched for published scientific studies on *T. gondii* and ELISA, and 57 articles were included. Overall, indirect (95%) and in-house (67%) ELISAs were the most used types of test among the studies examined, but the ‘ID Screen^®^ Toxoplasmosis Indirect Multi-species’ was common among commercially available tests. Varying diagnostic performance (sensitivity and specificity) and Kappa agreements were observed depending on the type of sample (serum, meat juice, milk), antigen (native, recombinant, chimeric) and antibody-binding reagents used. Combinations of recombinant and chimeric antigens resulted in better performance than native or single recombinant antigens. Protein A/G appeared to be useful in detecting IgG antibodies in a wide range of animal species due to its non-species-specific binding. One study reported cross-reactivity, with *Hammondia hammondi* and *Eimeria* spp. This is the first systematic review to descriptively compare ELISAs for the detection of *T. gondii* antibodies across different animal species.

## 1. Introduction

*Toxoplasma gondii* (Apicomplexa: Sarcocystidae) is an intracellular parasite that can infect endothermic animal species, including mammals and birds, with a worldwide distribution [1,2]. Infection with *T. gondii* also affects nearly one-quarter of the human population, making it an important zoonotic problem globally [3]. In most immunocompetent individuals, toxoplasmosis remains asymptomatic and self-limiting [4,5]; however, the infection can lead to significant morbidity and even mortality [6,7]. In livestock, infection with *T. gondii* can cause serious reproductive complications, including abortion, congenital deformity, stillbirth and foetal mummification [7,8,9], leading to significant economic losses [6,7]. Furthermore, *T. gondii* is an emerging threat to the health and welfare of wildlife populations worldwide [10]. For example, New World monkeys and Australian marsupials are thought to be highly susceptible to toxoplasmosis, often resulting in clinical disease and even death [1,11]. A wide range of clinical signs, including sudden death, encephalitis, lymphadenopathy, respiratory distress, interstitial pneumonia, and neurological signs have been reported in wild animals [12,13,14,15].

*Toxoplasma gondii* has a complex life cycle, with the sexual phase occurring in the definitive host (i.e., cats and other felids) and the asexual phase in intermediate hosts (i.e., humans as well as virtually all warm-blooded animals) [16]. Three obvious parasitic stages can be identified in the life cycle: sporozoites within sporulated oocysts, tachyzoites and bradyzoites, all of which can infect both definitive and intermediate hosts [17]. Upon primary infection in felids by the ingestion of tissue cysts, bradyzoites invade intestinal cells, resulting in numerous asexual and sexual developmental stages, eventually forming millions of oocysts that are excreted in faeces [18]. Both definitive and intermediate hosts can be infected upon the ingestion of infective oocysts from contaminated water bodies, pasture, vegetation, or eating raw or undercooked meat including tissue cysts containing bradyzoites [5,18]. In both definitive and intermediate hosts, vertical transmission via transplacental and lactogenic routes has also been reported [1,5]. Upon the ingestion of oocysts by an intermediate host, bradyzoites transform into tachyzoites in the intestine, multiply rapidly and disseminate throughout the body, thereby infecting any kind of cell in the body eliciting a strong immune response in immunocompetent individuals [19,20]. Subsequently, tachyzoites transform into bradyzoites and form dormant tissue cysts in the intermediate host, with a preference for neural and muscular tissues [2].

The detection of *T. gondii* can be achieved through direct and indirect methods. Direct methods involve the identification of parasitic stages by microscopic examination, the detection of parasitic DNA in samples using polymerase chain reaction (PCR), or isolation of the parasite utilising bioassays [21]. However, while these direct tests can be highly specific, they generally have limited sensitivity because they rely on the presence of one of the three infective stages of the parasite in the tested sample [22]. The microscopic detection of oocysts in faecal, water or environmental samples has particularly low sensitivity and is time-consuming [23]. Molecular techniques such as PCR can be used for the detection of both acute infections [22,24], with parasitic DNA found in the bloodstream due to rapidly multiplying tachyzoites, and chronic infections, using tissue samples targeting cysts in muscle and nervous tissues, usually from deceased animals [25]. However, this, similar to histochemical techniques, can be limited by the low abundance and random distribution of tissue cysts and disseminating tachyzoites [25,26]. The cat bioassay is a highly sensitive and specific test and is considered the “gold standard” for the detection of *T. gondii*, because it relies on the shedding of oocysts in the faeces of cats who have been fed tissue cysts [19,27]. However, this method is expensive, time consuming, and poses ethical challenges, making it impractical for routine screening of larger samples [21]. Thus, indirect methods involving the serological detection of parasite-specific antibodies have become the routine test for the diagnosis of toxoplasmosis in both animals and humans [23,28].

Serological diagnosis of toxoplasmosis takes advantage of the persistent presence of specific antibodies in serum following exposure to the parasite. Upon primary exposure to the parasite, IgM antibodies are produced in immunocompetent animals, which are classically short-lived [29]. Subsequently, IgG antibodies appear and persist for years, providing a reliable serological marker for the detection of previous exposure to *T. gondii* [29,30]. Serological techniques are relatively inexpensive, require a small volume of the sample, and can be used in live animals [10,31,32]. Many serological techniques, including the Sabin–Feldman dye test (DT), modified agglutination test (MAT), direct agglutination test (DAT), indirect immunofluorescence test (IFAT), indirect hemagglutination assay (IHA), latex agglutination test (LAT), Western blot (WB), and enzyme-linked immunosorbent assay (ELISA) have been widely used to detect *T. gondii*-specific antibodies in animals and humans [23,33]. However, while each of these tests has certain advantages, they also have certain limitations. Among all these tests, ELISA appears to be the most reliable, practical, economical, and widely used test for the detection of exposure to *T. gondii* in animals [34,35,36]. Only a small volume of sample is required, and the assay can be semi-automated, thereby making it suitable for large-scale screening [37,38]. Moreover, ELISAs can differentiate between immunoglobulin classes and are, therefore, useful in determining the phase of infection [29]. ELISAs can be divided into four main types: direct, indirect, sandwich, and competitive. However, all types use a colorimetric technique to quantify the analyte of interest in a liquid sample based on an antigen–antibody reaction, with the antigen/antibody complex in an immobilised phase [39,40]. Different ELISAs use different types of antigen (native, recombinant, chimeric) and secondary antibodies/antibody binding reagents (species-specific conjugates, non-species-specific conjugates) to detect antibodies [7,23]. However, the choice of components may significantly influence test performance. Indicators of test performance include sensitivity, specificity, and overall agreement (usually indicated by the Kappa statistic) with a reference test [41,42,43]. Commercial ELISA kits for the detection of *T. gondii* antibodies in domesticated animals are available, making routine and large-scale screening more practical. Moreover, flexibility in adapting the ELISA technique to desired research interests (such as the evaluation of novel antigens/antibodies for the development of more accurate assays, and the comparison of the performance of different serological tests) have made “in-house” ELISAs widely popular [7,44,45].

This systematic review compares different ELISAs for the detection of *T. gondii* antibodies in animals and animal products, their individual components and protocols, and how these influence diagnostic performance. The review provides direction on how to overcome existing limitations to developing more reliable and accurate ELISAs for the detection of *T. gondii*-specific antibodies in a wide range of animals.

## 2. Results

### 2.1. Literature Search and Eligible Articles

**Figure 1:**
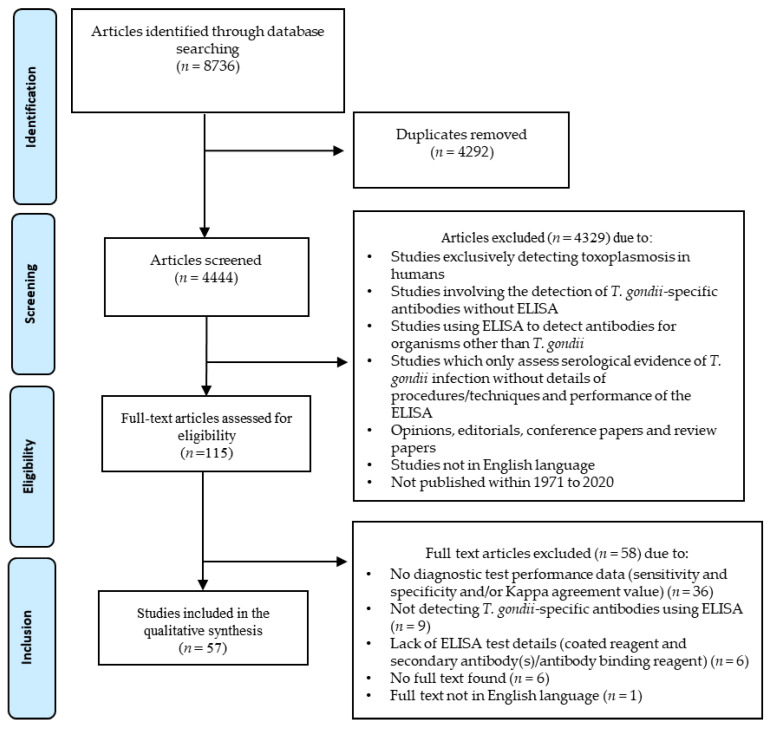
Preferred Reporting Items for Systematic Reviews and Meta-Analyses (PRISMA) flow diagram detailing the number of articles at each stage and the exclusion criteria applied.

During the literature search, 8736 studies were identified across four databases (Web of Science: *n* = 3179, Scopus: *n* = 2993, CAB Abstracts: *n* = 2201, and AGRICOLA: *n* = 363). Following the removal of duplicates (*n* = 4292), 4444 studies were subjected to title and abstract screening. Using exclusion and inclusion criteria, a total of 115 articles were selected for full-text evaluation, which resulted in the inclusion of 57 studies published between 1984 and 2020 (Figure 1).

### 2.2. General Characteristics of Studies Included in the Review

**Figure 2:**
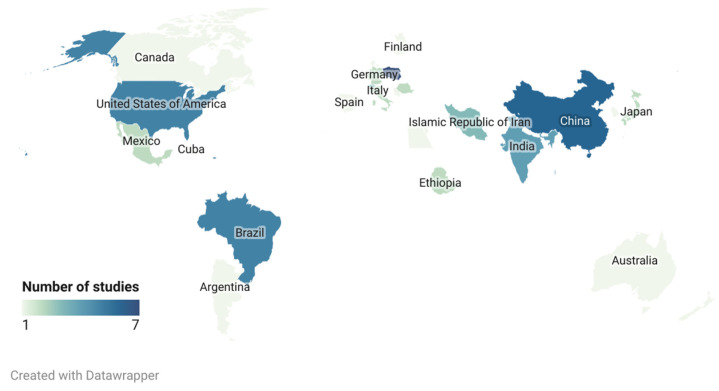
Geographical distribution of the studies (*n* = 57) included in the review. Detailed map can be accessed via https://datawrapper.dwcdn.net/MByRV/2/ (accessed on 30 March 2021).

Out of 57 studies describing the evaluation of diagnostic performance of different ELISAs for the detection of *T. gondii* antibodies, the majority originated from Europe (*n* = 21), followed by Asia (*n* = 16), South America (*n* = 9), North America (*n* = 6), Africa (*n* = 3) and Australia (*n* = 2) (Figure 2). Out of the 20 animal species covered, most of the studies (91%) focused on domesticated animals [33,37,46,47,48,49,50], but 7% were on zoo and wild animals [28,44,51,52], and one study targeted both domestic and wild animals [43]. Serum was the most widely used (89% of studies) sample for the detection of *T. gondii*-specific antibodies in animals, followed by meat juice/tissue fluid (7%) [37,45,53,54] and milk (4%) [55,56]. Most of the studies applied an indirect ELISA (95% of studies) [35,43,44,57,58] as the primary technique, with a smaller number using competitive ELISAs (3%) [59,60] or a combination of different methods (2%) (indirect IgG and IgM, blocking ELISA, reverse IgM capture ELISA) [29].

### 2.3. Type of Antigen Used in ELISAs

**Figure 3:**
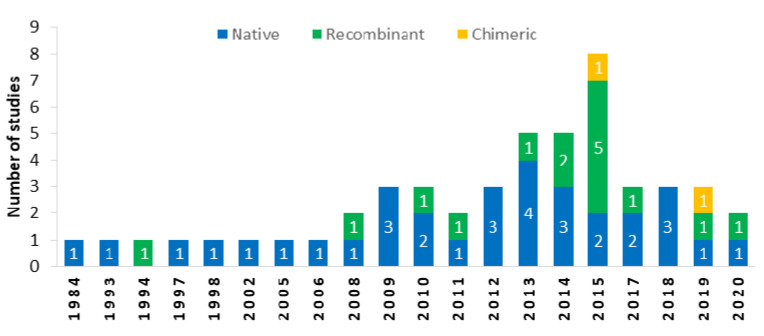
Frequency of antigen types used in ELISAs for the detection of *Toxoplasma gondii*-specific antibodies in animals over the study timeline. Native antigens (*n* = 33), recombinant (*n* = 15), and recombinant chimeric (*n* = 2).

Three main types of antigen, including native (66% of the 50 studies) [28,35,43,51,61], recombinant (30%) [24,44,50,57,62], and chimeric proteins (4%) [7,48], were used for the detection of *T. gondii*-specific antibodies in animals. Seven studies did not clearly indicate the type of antigen used, and hence were not included in the timeline below (Figure 3). Until approximately 2010, native antigens were most frequently used; however, the use of recombinant antigens became common in the last decade (Figure 3). Native antigens used in ELISAs consisted of either tachyzoite-based products or whole tachyzoites, whereas the six main types of recombinant antigens comprised surface antigens (SAG), dense granule proteins (GRA), microneme proteins (MIC), cyst matrix antigens (MAG), and rhoptry antigens (ROP), along with other peptide fragments.

### 2.4. Types of Antibodies Detected and Use of Secondary Antibodies/Antibody-Binding Reagents

Most of the studies (*n* = 52) focused on detecting *T. gondii*-specific IgG. Two studies detected specific IgM and IgG antibodies in pigs [29,63], where Lind et al. [29] used both an indirect IgM ELISA and a reverse IgM ELISA, while Terkawi et al. [63] only used an indirect IgM ELISA. Four studies tested *T. gondii*-specific IgY antibodies in birds, including three studies on chickens [33,47,64] and one in turkeys [62]. A variety of secondary antibodies or antibody-binding reagents were used. Based on their specificity for target species, these can be categorised into species-specific (74% of studies), multi-species (specific to a selected number of species) (10%), and non-species-specific (16%). A horseradish peroxidase (HRP) conjugate (not specified), which is a multi-species antibody-binding reagent, was used in both the ID Screen^®^ Toxoplasmosis Indirect Multi-species (IDvet, Grabels, France) and Pigtype^®^Toxoplasma Ab (Qiagen, Leipzig, Germany) commercial ELISA kits. Protein A and G, either separately or combined, have also been used as non-species-specific reagents.

### 2.5. Types of ELISAs Used

The majority of studies used in-house ELISAs (67%) [28,43,64,65] followed by commercial ELISAs (24%) [21,37,66,67] and modified commercial ELISAs (9%) [44,47,52,68,69]. In modified commercial ELISAs, anti-human secondary antibodies were replaced by animal-specific secondary antibodies.

Six different commercial ELISA kits were used, including the ID Screen Toxoplasmosis Indirect Multi-species (IDvet) (*n* = 6), PrioCHECK *Toxoplasma* Ab porcine ELISA (Thermo Scientific, Zurich, Switzerland) (*n* = 4), Chekit-Toxotest (IDEXX Laboratories, Bern, Switzerland) (*n* = 3), *Toxoplasma gondii* Antibody Test Kit (SafePath Laboratories, Carlsbad, CA, USA) (*n* = 2), Pigtype *Toxoplasma* Ab (Qiagen, Leipzig, Germany) (*n* = 1) and Toxo SPA-ELISA Kit (Haitai Bio, Shenzhen, China) (*n* = 1). The ID Screen^®^ Toxoplasmosis Indirect Multi-species (IDvet) ELISA kit uses the native P30 (SAG1) antigen and a multi-species HRP conjugate (not specified) to detect *T. gondii*-specific antibodies in multiple species. All other commercial kits use whole tachyzoite antigens, while no such information is available for the Chekit-Toxotest (IDEXX Laboratories). The PrioCHECK *Toxoplasma* Ab porcine ELISA (Thermo Scientific), Chekit-Toxotest (IDEXX Laboratories) and *Toxoplasma gondii* Antibody Test Kit (SafePath Laboratories) use species-specific secondary antibodies, whereas the Pigtype^®^*Toxoplasma* Ab (Qiagen) and Toxo SPA-ELISA kit (Haitai Bio) use a multi-species conjugate (not specified) and protein A, respectively, as secondary antibodies.

### 2.6. Diagnostic Performance

The diagnostic performance of different ELISAs was compared across different sample and antigen types, with the use of single vs. combinations of antigens and various antibody binding reagents. The following sections provide an overview of the diagnostic performance of ELISAs used for the detection of *T. gondii*-specific antibodies in animals.

#### 2.6.1. Milk and Meat Juice ELISAs

Out of the five studies that utilised milk and meat juice samples, the ID Screen Toxoplasmosis Indirect Multi-species (IDvet) ELISA kit achieved more than 97% sensitivity and specificity and excellent agreement (Kappa value 0.949) for milk compared with serum, using the same ELISA kit. The reported sensitivity and specificity values for milk ranged from 88.7 to 97.55% and 97.42 to 97.83%, respectively. For meat juice, on the other hand, sensitivity and specificity values ranged from 3.6 to 96.7% and 83.9 to 100%, respectively (Table 1). Three commercial ELISA kits, including the PrioCHECK *Toxoplasma* Ab porcine ELISA (Thermo Scientific), Pigtype^®^*Toxoplasma* Ab (Qiagen), and ID Screen^®^ Toxoplasmosis Indirect Multi-species (IDvet), reported better overall performance compared to other commercial kits using meat juice. The *Toxoplasma gondii* Antibody Test Kit (SafePath Laboratories) showed low sensitivity (3.6%), with slight agreement (Kappa value 0.05) with the MAT test [37]. However, the same ELISA kit reported a better sensitivity (88.6%) in another study using meat juice [45]. In-house ELISAs reported higher sensitivity and specificity values of 96.7% and 100%, respectively [54].

#### 2.6.2. Use of Single Recombinant Antigens

Recombinant antigens are specific immunogenic proteins produced in bacterial or appropriate eukaryotic systems using recombinant DNA technology, which are also used for the immunodetection of *T. gondii* infection [44,70]. Twelve different recombinant antigens across four major categories (surface granular antigens (SAG), dense granular proteins (GRA), microneme proteins (MIC) and peptide fragments) were used in a range of animal species (Table 2). Dense granular proteins (GRA) were the most frequently used recombinant antigens, with GRA7 being the most common (*n* = 8). Half of the studies using GRA7 reported excellent agreement with respective reference tests and high sensitivity (84.2–100%) and specificity (91.6–99.1%) (Table 2) [33,34,44,65]. Two studies claimed higher than 85% sensitivity and specificity, with substantial agreement [63,71], whereas one study reported a lower sensitivity and only fair agreement [46]. All studies using GRA1 (*n* = 4) reported higher than 75% sensitivity and specificity, and substantial agreement with the reference test. GRA2, GRA6, GRA14 and GRA15 were used less frequently (*n* = 1 per each antigen), the use of GRA6 and GRA14 resulted in higher than 80% sensitivity and substantial agreement with the reference tests, while GRA2 and GRA15 resulted in less than 30% sensitivity and fair to slight agreement with the reference tests, respectively.

Among SAG antigens, SAG 2 was used more frequently (*n* = 5) and with higher sensitivity (80–91.89%) and specificity (85.71–91.43%) than SAG 1 (*n* = 3; higher than 83% sensitivity and specificity). The least commonly used antigens were MIC and peptide fragments. Zhang et al. [58] used MIC3 in two host species and obtained excellent agreement. In contrast, the use of MIC10 achieved only low sensitivity and slight agreement [46]. Among recombinant polypeptide proteins used in cats, H4 performed better than H11 [72].

#### 2.6.3. Use of Combinations of Recombinant Antigens

Seventeen different recombinant antigen combinations (M1–M17), containing between two and five antigens, were used (Table 3). Two additional categories of recombinant antigen, cyst matrix antigen (MAG), and rhoptry antigen (ROP), which were not used in single recombinant antigen ELISAs, were used in recombinant antigen combinations. Out of seventeen different combinations, nine combinations (M1, M2, M3 in jaguars, M4, M7, M8, M9, M14 in sheep and M15) resulted in the strongest performance, with greater than 90% sensitivity and specificity. Antigens from the SAG and/or GRA categories were widely used among combinations. Either SAG or GRA antigens were included in sixteen out of seventeen combinations (all combinations except M1). Moreover, combinations of both SAG and GRA antigens were used in seven out of seventeen instances (M2, M3, M4, M5, M6, M14, M17), which resulted in good sensitivity (77.8–100%) and specificity (84.4–100%). Furthermore, certain recombinant antigen mixtures, including M1 (H4 + H11), M2 (SAG1 + GRA7), M3 (SAG1 + GRA7), M5 (SAG2 + GRA6), M6 (SAG2 + GRA7), M10 (GRA2 + GRA7), M11 (GRA6 + GRA), M16 (GRA2 + GRA6+ GRA7 + GRA15) and M17 (SAG2 + GRA2 + GRA6 + GRA7 + GRA15), had better diagnostic performances (either sensitivity or specificity or both/Kappa values) than if being used as single recombinant antigens (Table 2) in the same host species. Moreover, variable diagnostic performance was observed when the same antigen combination was used in different animal species (M3, M13, M14, M15).

#### 2.6.4. Recombinant Chimeric Antigens

Chimeric antigens are a new generation of recombinant antigens and have only been used in two studies. They are made by the fusion of two or more fragments of well-known antigens, hence containing multiple immunoreactive epitopes from each antigen. Nine different combinations of such recombinant chimeric antigens were reported for use in horses, sheep, goats, and pigs (Table 4), but none of the studies reported the level of Kappa agreement with their respective reference test. Of the nine chimeric antigens used, CM5 (SAG2-GRA1-ROP1L) and CM8 (AMA1-SAG2-GRA1-ROP1) were the most effective, with high sensitivity (93.8–100% and 95.56–97.92%, respectively) and specificity (100% in both). Both CM5 and CM8 comprised fragments from SAG, GRA, ROP antigens, and CM8 additionally included fragments of apical membrane antigen (AMA). The remaining chimeric antigens varied in test performance, with sensitivity ranging from 28.4 to 100% and specificity ranging from 95.12 to 100%, depending on the animal species tested. All chimeric antigens were reported to have high specificity of more than 95% across all animal species tested.

#### 2.6.5. Comparison of Native and Recombinant/Chimeric Antigens

Nine different studies compared the diagnostic performance of native and recombinant/chimeric antigens in eight species, resulting in twelve comparisons provided here (Table 5). Native antigens were either lysate antigens from whole tachyzoites (TLA) or soluble antigens (TSA) of *T. gondii*. Only the most effective recombinant antigen or antigen combination from each study (according to the authors of each study when multiple recombinant antigens or combinations were used) were included in this comparison. Most comparisons (8/12) reported similar or slightly higher sensitivity for recombinant and chimeric antigens (84.2–100%) compared to native antigens (68.4–100%). However, in four instances, native antigens produced slightly better sensitivity than a recombinant antigen combination: M17, M1 and two chimeric antigens, CM5 in pigs and CM8 in goats (Table 5). Recombinant and chimeric antigens reported overall better specificity than native antigens. Eleven out of 12 comparisons reported similar or higher specificity in recombinant/chimeric antigens, with values ranging from 95.36 to 100%. One study reported slightly higher specificity (99.3%) for the native antigen compared to GRA7 (92.5%) [65]; however, three other comparisons achieved higher specificity using GRA7 than with native antigens [33,34,71].

#### 2.6.6. Diagnostic Performance of Non-Species-Specific Antibody Binding Reagents

Non-species-specific secondary antibody binding reagents have the advantage of being able to detect antibodies across a broad range of hosts without the need for species-specific conjugates. In this review, 16% of studies used the non-species-specific reagents protein A, protein G, and protein A/G, targeting eleven different species of animals and using indirect ELISAs (Table 6). Protein A/G was used in five different studies across 11 different mammalian species, with all of them (except one study where the Kappa value was not given) reporting substantial to excellent agreement with their reference test. Sensitivity and specificity of using protein A/G were 68.4–92% and 89–99.1%, respectively. Protein A was used in four different studies in three host species, and agreement with the reference test varied from moderate to excellent, reporting sensitivity and specificity values of 89.5–100% and 82–100%, respectively.

Protein G was only used in one study, across four different species, producing substantial to excellent agreement with IHA as the reference test, and varying sensitivity (72–97%) and specificity (95–100%).

#### 2.6.7. Cross-Reactivity of ELISAs Used for Animals

Eleven studies investigated, or mentioned, the potential cross-reactivity of antigens (Table 7). *Neospora caninum* was the most tested organism for cross-reactivity (*n* = 7), but none of the studies reported cross-reactions with that species. Several other studies tested other organisms, including *Sarcocystis* spp., *Besnoitia* spp., *Isospora suis*, *Trichinella* spp., *Ascaris suum*, *Salmonella* spp., *Yersinia* spp. and *Actinobacillus* spp., but did not detect cross-reactivity with *T. gondii*. Based on the studies included in this review, cross-reactivity was only observed with *Hammondia hammondi* and *Eimeria* spp. in experimentally infected turkeys when a recombinant antigen combination M12 (GRA7 and GRA8) was used [62].

## 3. Discussion

ELISA is one of the most effective serological techniques used to detect exposure to *T. gondii* in animals [23,43]. This review evaluated 57 articles describing the performance of different ELISAs in detecting *T. gondii* antibodies in 20 different animal species. To the best of our knowledge, this is the first systematic review to provide a descriptive comparison on the performance of different ELISAs for the detection of *T. gondii* antibodies across different animal species. The results highlight the potential opportunities for refinements in ELISAs to be used in animals, including wildlife.

Researchers used different reference tests to estimate and compare the diagnostic performance of their ELISAs. Given that no perfect diagnostic test exists to detect toxoplasmosis in animals, each reference test is likely to differ in sensitivity and specificity [78], thereby affecting the calculated performance of the ELISA in question. Hence, comparison of diagnostic performance across different studies is complex and some seemingly perfect results (100% sensitivity and/or specificity) should be interpreted with care because perfect results may not necessarily mean the ELISA is perfect. In addition, sample sizes varied across studies and study power should be taken into consideration in serological comparison studies. The sample size is positively related to statistical power, and sufficiently large sample sizes are important in obtaining more accurate, valid, and reliable results [47,79].

There are clear, practical advantages to being able to use a single ELISA kit across multiple species and different types of sample. The most commonly used commercial kit, ID Screen^®^ Toxoplasmosis Indirect Multi-species (IDvet, France), uses native P30 (SAG1) antigen and anti-multi-species conjugate as the secondary antibody, which makes it suitable for the detection of *T. gondii*-specific antibodies in ruminant, swine, dog and cat serum, but also in milk and meat juice [37]. It may subsequently be useful for the detection of *T. gondii* antibodies in wild ruminants, porcine species, canids, and felids. Moreover, the use of SAG 1 antigen in this kit might provide greater specificity than tests using whole tachyzoite antigen [49]. Thus, future validation of this test in a range of wildlife and other animal species suspected to be infected with *T. gondii* could be worthwhile.

The indirect ELISA was the most commonly used method in the studies included in the review. Indirect ELISAs involve two antibody-binding steps: first, primary antibodies in the sample bind to the immobilised coated antigen, and then labelled secondary antibodies bind to the primary antibodies, allowing signal amplification and identification of the primary antibody of interest [80]. A wide range of secondary antibodies are commercially available for most domesticated animal species, making the indirect ELISA method versatile for use in those species. An advantage of ELISAs over other serological methods is that they can be used for the detection of a range of immunoglobulin classes, including IgG, IgM, IgA, IgD and IgE [81,82,83,84]. However, most studies focused on animals only aim to detect mammalian IgG and IgM, and avian IgY antibodies (which resemble mammalian IgG). In fact, most studies (*n* = 52) focused on the detection of IgG or did not discriminate between the classes of antibodies. Only two studies specifically reported the detection of IgM [29,63]. IgM antibodies are often short-lived (2–4 weeks) and classically regarded as a marker for acute infection [85]. However, IgM antibodies can persist from a few months to a year, meaning that positive IgM results alone are not sufficient to discriminate between phases of infection [86]. However, testing IgM levels is useful when coupled with other diagnostic tests (e.g., IgG avidity testing, polymerase chain reaction) and widely used to determine the phase of infection in pregnancy-associated toxoplasmosis in humans [86,87]. IgG antibodies often persist for life in immunocompetent individuals, providing a reliable marker after primary infection, suggesting that a switch from isotype IgM to IgG has occurred, and they detect the chronic phase of *T. gondii* exposure in both animals and humans [29,30,85].

Based on the overall data reviewed, recombinant and chimeric antigens resulted in similar or better diagnostic performance than native *T. gondii* antigens. *Toxoplasma gondii* tachyzoite-based native antigens have commonly been used across many serological tests, including ELISA [23]. However, recombinant and chimeric antigens possess other advantages over native antigens, including the ease of production, reduced cost, and less exposure to biohazardous procedures [88]. Moreover, assay optimisation and standardisation are easier and researchers have the freedom to precisely construct the required antigen composition of interest [48,88]. However, there are some disadvantages, including inefficient expression and misfolding of proteins during the production process inside the prokaryotic systems, which could affect the affinity of the assay [89].

This review identified five well-defined categories of recombinant antigens (SAG, GRA, MIC, MAG, ROP), and members of each category were used as single antigens, or in a combination. Except for M1 (H4 + H11), all recombinant antigen combinations contained SAG and/or GRA antigens, indicating their wide use. Among single recombinant antigens, SAG and GRA were most frequently used. SAG1 and SAG2 are highly immunodominant and abundant antigens, present in the tachyzoite stage [90,91,92]. Moreover, SAG1 and SAG2 are highly conserved among different *T. gondii* strains and can be detected in both acute and chronic infections, thereby improving their value as diagnostic markers [57,91,93]. Furthermore, in human studies, both SAG1 [91,94] and SAG2 [93,95,96] have successfully been used to detect *T. gondii*-specific antibodies.

Dense granular proteins (GRA) are involved in *T. gondii*’s replication inside the host cells and are secreted by both tachyzoites and bradyzoites [97]. GRA7 is well-expressed on both the surface and within the cytoplasm of the infected host cell, resulting in direct exposure to the host’s immune system and provoking a strong immune response in both acute and chronic toxoplasmosis [98,99]. Overall, this antigen performed better than several other GRAs across several host species, although one study reported poor sensitivity (35.1%) and only fair agreement with the reference test (LAT). However, while GRA7 was reported to have higher than 80% sensitivity and specificity, other members of the GRA family were not widely reported among studies.

Combinations of recombinant antigens tended to perform better than single recombinant antigens. The performance of single recombinant antigens is likely variable because they do not represent the same complex of epitopes as seen in native antigens [70]. Infected hosts mount varying humoral immune responses depending on the infective stage of the parasite; thus, a single recombinant antigen is probably not capable of binding with all stage-specific antibodies [70]. In contrast, a combination of different recombinant antigens may allow *T. gondii*-specific antibodies to recognise multiple epitopes from different parasitic stages [48,70]. Based on the diagnostic performance reported, the combination of SAG and GRA antigens, including SAG1, SAG2, and GRA7, may provide better diagnostic performance.

Only two studies reported the use of the novel generation of chimeric antigens. Similar to combinations of recombinant antigens, the enhanced epitope complexity of chimeric antigens probably results in recognition of different parasitic stages [7,48]. Thus, stage-specific *T. gondii* antibodies can be identified in the serum of the infected host, improving the sensitivity of the assay [7,48]. CM5 (SAG2-GRA1-ROP1L) and CM8 (AMA1-SAG2-GRA1-ROP1) performed better than other chimeric antigens. Despite the promising results reported to date, the diagnostic performance of chimeric antigens should be evaluated compared to combinations of recombinant antigens across many species to understand the broader value of this new, promising serological tool. Nevertheless, chimeric antigens have been successfully used as vaccine candidates [100] and to detect *T. gondii* infection in humans [101], and as a diagnostic tool to detect other pathogenic infections in humans [102,103,104].

In this review, three different categories of secondary antibodies/antibody binding reagents were identified based on their specificity to target species. Species-specific secondary antibodies were widely used in the studies [49,64,71]. This is likely a consequence of the fact that most of the studies focused on common domesticated animals, for which a wide range of species-specific or taxon-specific secondary antibodies are commercially available. However, the availability of specific-specific secondary antibodies is limited for other animals, such as wildlife species [10,43]. Subsequently, researchers have used multispecies and non-species-specific reagents to overcome this problem. Multispecies conjugates were used in two commercial ELISA kits, including the commercially available ID Screen^®^ Toxoplasmosis Indirect Multi-species (IDvet) and Pigtype^®^Toxoplasma Ab (Qiagen) ELISA kits. However, data about the exact reagents present in those two kits were not available. Protein A/G combinations or protein A and G separately were used in several studies as non-species-specific antibody binding reagents. Both protein A and G are bacterial proteins that are capable of binding with the Fc region of the mammalian IgG [105], but appear to have poor binding capability for bird and reptilian antibodies [106]. Protein A/G combines the IgG binding capabilities of both protein A and G, and can therefore be used as a reliable serological tool for the detection of IgG antibodies across a wide range of mammal species [43,106]. Nonetheless, varying results in the binding capability of these non-species-specific reagents with IgG can be expected among different target host species. This might be due to slight variations in both binding ability and structure of the binding domains of the IgG as a result of genetic variation between species [43]. Hence, prior to use in an immunoassay, assessment of immunoglobulin binding capability of non-species-specific reagents with the target species is advisable [107,108].

Cross-reactivity between *T. gondii* antigens and antibodies against other organisms can reduce the specificity of serological assays [43]. Only one study, using the antigen M12 (GRA7 and GRA8), reported cross-reactivity (with *Hammondia hammondi* and *Eimeria* spp.) [62]. The apicomplexan parasites *T. gondii* and *H. hammondi* have structural similarities; cats act as a definitive host for both parasites [109]. Antigenic similarities between both parasites and the presence of cross immunity in infected hosts have been reported in other studies [109,110]. Thus, caution should be exercised when selecting the antigen, and ELISAs should be evaluated for possible cross-reactivity to optimise specificity [43].

Despite the effort to obtain and include all available literature related to this review, publication bias may have influenced the selection of studies, and some studies which were of low quality, unavailable, or in other languages were not included. Additionally, this review has provided a narrative synthesis of results, and statistical tests to compare results across various studies were not performed. Studies used different reference tests for comparison with ELISA performance; therefore, we did not evaluate the performance of such reference tests. Thus, future studies might consider statistical modelling approaches to standardise the performance of reference tests.

## 4. Materials and Methods

### 4.1. Review Protocol

This systematic review was reported according to the Preferred Reporting Items for Systematic Reviews and Meta-Analyses (PRISMA) guidelines (http://www.prisma-statement.org/, accessed on 30 March 2021). The review protocol was registered in an international prospective register of systematic reviews (PROSPERO) with the registration ID (CRD42020208925).

### 4.2. Search Strategy

A systematic search in four online scientific databases (Web of Science, Scopus, CAB Abstracts, Agricola) was conducted. The search strategy involved the use of Boolean operators with the following search terms: (“*Toxoplasma*” OR “Toxoplasmosis” OR “*T. gondii*” OR “*Toxoplasma gondii*” OR “Toxoplasm*”) AND (“ELISA” OR “enzyme linked immunosorbent assay” OR “*ELISA”). Only studies published in English and between 1971 and 2020 (since ELISA was developed, in 1971) were included [111].

### 4.3. Quality Assessment and Selection

Citations and abstracts were exported into EndNote X9 (Clarivate Analytics, Philadelphia, PA, USA) and all duplicates were removed. Subsequently, title and abstract screening was performed to select relevant articles based on the following exclusion criteria: (1) studies exclusively detecting toxoplasmosis in humans; (2) studies involving the detection of *T. gondii*-specific antibodies in animals using techniques other than ELISA; (3) studies using ELISA to detect antibodies in animals for organisms other than *T. gondii*; (4) studies which only assessed the serological evidence of toxoplasmosis without providing the details of procedures/techniques and diagnostic performance; and (5) opinions, editorials, conference papers, and review papers. Selected articles were then read in full to review their eligibility for inclusion. In addition to the above-mentioned exclusion criteria, studies without details of antigen-coated/immobilised agent and secondary antibody/antibody binding reagent were excluded. Studies providing information on diagnostic performance, using either sensitivity and specificity of the ELISA or Kappa agreement values, were included (Figure 1).

### 4.4. Data Extraction and Analyses

Data extraction was performed by the first author (K.L.D.T.D.L.). Any discrepancy was resolved by discussion with co-authors, who acted as secondary reviewers where required. The following data were extracted from eligible studies and documented in an Excel spreadsheet: species, sample type (serum, meat juice, milk) and size, animal category (domesticated, laboratory, wild and zoo), nature of the ELISA (in-house, commercial), ELISA type (direct, indirect, sandwich, competitive, other modifications, e.g., reverse), type of antigen(s) used (native, recombinant, chimeric), secondary antibody/immunoglobulins binding reagent, antibodies detected (IgG or IgM or both), the total number of samples tested and the number of positives, sensitivity and specificity of the ELISA, agreement (Kappa value) of the ELISA with the reference test, and cross-reactions. Animal types were categorised as follows: companion and livestock animals as domesticated animals; mice, hamsters, and guinea pigs as lab animals; free-ranging, undomesticated, and zoo animals as wild and zoo animals. All Kappa agreement values reported in the included studies were interpreted as follows: less than chance (≤0), slight (0.01–0.20), fair (0.21–0.40), moderate (0.41–0.60), substantial (0.61–0.80), and excellent (≥0.81) [41].

Relevant manufacturers were contacted to obtain technical data for their ELISA kits where required. Descriptive analyses of extracted data were performed, which are presented together with a narrative synthesis in this review. The bar chart and map were created using Microsoft Excel and Datawrapper.

## 5. Conclusions

In conclusion, most ELISAs used to detect exposure to *T. gondii* in animals were of the indirect type, and they generally used serum and targeted *T. gondii*-specific IgG. In-house ELISAs were most popular; however, among commercial kits, the ID Screen^®^ Toxoplasmosis Indirect Multi-species (IDvet, France) kit appeared to be effective due to its better performance and utility across multiple species as well as the possibility of testing different types of samples (serum, milk, or meat juice). Recombinant antigen combinations and chimeric antigens overall provided better diagnostic performance than native antigens or single recombinant antigens. A wide range of secondary antibodies are commercially available for domestic animals, but for species where no secondary antibodies are available, protein A/G can provide an alternative solution. Cross-reactivity with *T. gondii*-related parasites should be considered to improve the diagnostic performance of the assay. The findings of this study can be used to overcome existing limitations and develop new and reliable serological assays for the detection of *T. gondii* antibodies in a range of animal species. In the future, updating this review including both animal and human studies with a combination of age, gender groups, and other diagnostic methods with statistical evaluation would provide a better understanding of the detection of *T. gondii* infection worldwide.

## Figures and Tables

**Table 1:** Performance of different enzyme-linked immunoassays (ELISAs) in detecting *Toxoplasma gondii*-specific antibodies in milk and meat juice/tissue fluid samples.

Sample Type	ELISA	Host Species	Positive % (n/N)	Se (%)	Sp (%)	Agreement (Kappa Value)	Reference Test Used	Reference
Milk	In-house	Goat	20 (120/600)	88.7	97.4	ND	MAT (serum and milk)	[55]
	ID Screen^®^ Toxoplasmosis Indirect Multi-species (IDvet)	Goat	59 (59/100)	97.55	97.8	0.949	Same commercial ELISA with serum	[56]
Meat Juice/Tissue fluid	PrioCHECK *Toxoplasma* Ab porcine ELISA(Prionics)	Pig	41.1 (37/90)	96.4	83.9	0.74	MAT	[37]
	Pigtype^®^*Toxoplasma* Ab (Qiagen)	Pig	27.8 (25/90)	89.3	100	0.92	MAT	[37]
	ID Screen^®^ Toxoplasmosis Indirect Multi-species (IDvet)	Pig	24.4 (22/90)	78.6	100	0.83	MAT	[37]
	*Toxoplasma gondii* Antibody Test Kit (SafePath Laboratories)	Pig	1.1 (1/90)	3.6	100	0.05	MAT	[37]
	*Toxoplasma gondii* Antibody Test Kit (SafePath Laboratories)	Pig	88.5 (62/70)	88.6	98	ND	Mouse bioassay	[45]
	In-house	Pig	6.2 (60/969)	96.7	100	ND	Commercial ELISA Kit	[54]

n—test positive; N—number of samples tested; Se—Sensitivity; Sp—Specificity; MAT—modified agglutination test; ND—no data.

**Table 2:** Comparison of sensitivity, specificity, and level of agreement with a reference test for different single recombinant antigen-based enzyme-linked immunoassays (ELISAs) to detect serum antibodies to *Toxoplasma gondii* in various animals.

Antigen Category	Antigen	Positive % (n/N)	Host Species	Se (%)	Sp (%)	Agreement (Kappa Value)	Reference Test Used for Comparison	Reference
Surface antigens (SAG)	SAG1	75 (39/52)	Jaguar	92.5	83.3	0.74	Commercial ELISA (TLA)	[44]
	SAG1	71.8 (181/252)	Cattle	84.38	87.9	0.73	IFAT	[24]
	SAG1	44.6 (25/56)	Goat	83.3	84.4	ND	MAT	[73]
	SAG2	ND	Cat	91.89	88.1	0.67	LAT	[46]
	SAG2	41.26 (26/63)	Goat	82.14	91.4	0.741	IFAT	[57]
	SAG2	50 (30/60)	Sheep	81.25	85.7	0.667	IFAT	[57]
	SAG2	64.44 (29/45)	Cattle	87.1	85.7	0.701	IFAT	[57]
	SAG2	61.5 (115/258)	Cattle	80	88.6	0.689	IFAT	[50]
Dense granule proteins (GRA)	GRA1	15.3 (20/131)	Mink	78.9	95.5	0.73	WB	[34]
	GRA1	75 (39/52)	Jaguar	92.5	83.3	0.74	Commercial ELISA (TLA)	[44]
	GRA1	16.4 (18/110)	Chicken	81.3	94.7	0.72	WB	[33]
	GRA1	16.2 (42/259)	Dog	81	95.4	0.66	ELISA (TLA)	[71]
	GRA2	ND	Cat	27.3	96.52	0.3	LAT	[46]
	GRA6	ND	Cat	82.43	88.7	0.62	LAT	[46]
	GRA7	ND	Cat	35.1	89.9	0.27	LAT	[46]
	GRA7	21.6 (40/185)	Cat	89.7	92.5	0.92	IFAT/MAT	[65]
	GRA7	13 (17/131)	Mink	84.2	99.1	0.83	WB	[34]
	GRA7	76.9 (40/52)	Jaguar	97.5	91.6	0.89	Commercial ELISA (TLA)	[44]
	GRA7	15.5 (17/110)	Chicken	100	98.9	0.96	WB	[33]
	GRA7	55.9 (33/59)	Pig	90.63	85.2	0.76	LAT	[63]
	GRA7	42.8 (24/56)	Goat	80	84.4	ND	MAT	[73]
	GRA7	16.2 (42/259)	Dog	91	97.7	0.8	ELISA (TLA)	[71]
	GRA14	47.4 (28/59)	Pig	81.25	92.6	0.73	LAT	[63]
	GRA15	ND	Cat	17.57	86.4	0.04	LAT	[46]
Microneme proteins (MIC)	MIC3	41.1 (81/197)	Pig	ND	ND	0.86	MAT	[58]
	MIC3	45.8 (11/24)	Dog	ND	ND	0.85	MAT	[58]
	MIC10	ND	Cat	16.21	85.8	0.02	LAT	[46]
Other peptide fragments	H4	25.81 (79/306)	Cat	93	100	ND	DAT, IFAT, DT	[72]
	H11	12.41 (38/306)	Cat	64	100	ND	DAT, IFAT, DT	[72]

n—test positive; N—number of samples tested; Se—Sensitivity; Sp—Specificity; MAT—modified agglutination test; IFAT—indirect fluorescent antibody test; WB—Western blot; LAT—latex agglutination test; DAT—direct agglutination test; TLA—*Toxoplasma gondii* lysate antigen; ND—no data.

**Table 3:** Comparison of sensitivity, specificity, and level of agreement with a reference test for different recombinant antigen combinations based enzyme-linked immunoassays (ELISAs) to detect serum antibodies to *Toxoplasma gondii* in various animals.

Combination of Antigens	Antigens	Positive % (n/N)	Host Species	Se (%)	Sp (%)	Agreement (Kappa Value)	Reference Test Used for Comparison	Reference
M1	H4 + H11	31.37 (96/306)	Cat	95	100	ND	DAT, DT, IFAT	[72]
M2	SAG1 + GRA1	75 (39/52)	Jaguar	95	91.6	0.84	Commercial ELISA (TLA)	[44]
M3	SAG1 + GRA7	76.9 (40/52)	Jaguar	97.5	91.6	0.89	Commercial ELISA (TLA)	[44]
		46.4 (26/56)	Goat	86.6	84.4	ND	MAT	[73]
M4	SAG2 + GRA1	81.5 (88/108)	Sheep	100	95	ND	ND	[70]
M5	SAG2 + GRA6	ND	Cat	94.59	89.6	0.72	LAT	[46]
M6	SAG2 + GRA7	ND	Cat	90.54	85.5	0.62	LAT	[46]
M7	SAG2 + ROP1	81.5 (88/108)	Sheep	100	95	ND	ND	[70]
M8	GRA1 + ROP1	81.5 (88/108)	Sheep	100	100	ND	ND	[70]
M9	GRA1 + GRA7	76.9 (40/52)	Jaguar	97.5	91.6	0.89	Commercial ELISA (TLA)	[44]
M10	GRA2 + GRA7	ND	Cat	44.59	89.3	0.35	LAT	[46]
M11	GRA6 + GRA7	ND	Cat	74.32	89	0.58	LAT	[46]
M12	GRA7 + GRA8	20.2 (387/1913)	Turkey	92.6–100	78.1–100	ND	ND	[62]
M13	SAG1 + MIC1 + MAG1	37.21 (32/86)	Horse	88.9	100	ND	DAT, IFAT	[7]
		57.07 (109/191)	Sheep	77.9	92.2	ND	DAT, IFAT	[7]
		42.86 (72/168)	Pig	88.9	100	ND	DAT, IFAT	[7]
M14	SAG2 + GRA1 + ROP1	32.56 (28/86)	Horse	77.8	100	ND	DAT, IFAT	[7]
		73.30 (140/191)	Sheep	100	100	ND	DAT, IFAT	[7]
		39.29 (66/168)	Pig	81.5	100	ND	DAT, IFAT	[7]
		81.5 (88/108)	Sheep	100	100	ND	ND	[70]
M15	GRA1 + GRA2 + GRA6	36.05 (24/86)	Horse	66.7	100	ND	DAT, IFAT	[7]
		70.16 (129/191)	Sheep	92.1	100	ND	DAT, IFAT	[7]
		46.43 (44/168)	Pig	54.3	100	ND	DAT, IFAT	[7]
M16	GRA2 + GRA6+ GRA7 + GRA15	ND	Cat	70.27	86.1	0.5	LAT	[46]
M17	SAG2 + GRA2 + GRA6 + GRA7 + GRA15	ND	Cat	89.19	95.4	0.81	LAT	[46]

n—test positive; N—number of samples tested; Se—Sensitivity; Sp—Specificity; MAT—modified agglutination test; IFAT—indirect fluorescent antibody test; WB—Western blot; LAT—latex agglutination test; DAT—direct agglutination test; DT—Dye test; TLA—*Toxoplasma gondii* lysate antigen; ND—no data.

**Table 4:** Comparison of sensitivity and specificity values for different recombinant chimeric antigen-based enzyme-linked immunoassays for the detection of antibodies to *Toxoplasma gondii* in various animals.

	Combination of Chimeric Recombinant Antigens	Species	Positive % (n/N)	Se (%)	Sp (%)	Reference Test	Reference
CM1	GRA1-GRA2-GRA6	Horse	36.1 (31/86)	86.1	100	DAT, IFAT	[7]
		Sheep	70.2 (134/191)	95.7	100	DAT, IFAT	
		Pig	46.4 (78/168)	96.3	100	DAT, IFAT	
CM2	MIC1-MAG1-SAG1s	Horse	31.4 (27/86)	75	100	DAT, IFAT	
		Sheep	71.7 (137/191)	97.9	100	DAT, IFAT	
		Pig	22.0 (37/168)	45.7	100	DAT, IFAT	
CM3	SAG1L-MIC1-MAG1	Horse	32.6 (28/86)	77.8	100	DAT, IFAT	
		Sheep	73.3 (140/191)	100	100	DAT, IFAT	
		Pig	43.5 (73/168)	90.1	100	DAT, IFAT	
CM4	SAG2-GRA1-ROP1S	Horse	21 (18/86)	50	100	DAT, IFAT	
		Sheep	73.3 (140/191)	100	100	DAT, IFAT	
		Pig	13.7 (23/168)	28.4	100	DAT, IFAT	
CM5	SAG2-GRA1-ROP1L	Horse	41.9 (36/86)	100	100	DAT, IFAT	
		Sheep	73.3 (140/191)	100	100	DAT, IFAT	
		Pig	45.2 (76/168)	93.8	100	DAT, IFAT	
CM6	AMA1N-SAG2-GRA1-ROP1	Sheep	Not clearly mentioned	97.9	97.62	LAT, IFAT	[48]
		Goat	Not clearly mentioned	88.9	100	LAT, IFAT	
CM7	AMA1C-SAG2-GRA1-ROP1	Sheep	Not clearly mentioned	95.8	95.24	LAT, IFAT	
		Goat	Not clearly mentioned	95.6	97.56	LAT, IFAT	
CM8	AMA1-SAG2-GRA1-ROP1	Sheep	Not clearly mentioned	97.9	100	LAT, IFAT	
		Goat	Not clearly mentioned	95.6	100	LAT, IFAT	
CM9	SAG2-GRA1-ROP1-GRA2	Sheep	Not clearly mentioned	97.9	97.62	LAT, IFAT	
		Goat	Not clearly mentioned	57.8	95.12	LAT, IFAT	

n—number of samples tested positive; N—total number of samples tested; Se—Sensitivity; Sp—Specificity; IFAT—indirect fluorescent antibody test; LAT—latex agglutination test; DAT—direct agglutination test.

**Table 5:** Comparison of sensitivity and specificity between native and recombinant/chimeric antigen(s) in detecting *Toxoplasma gondii*-specific antibodies in multiple animal species.

Species	Antigen (s)	Se (%)	Sp (%)	Reference
Cat	M17 (SAG2 + GRA2 + GRA6 + GRA7 + GRA15)	89.19	95.36	[46]
	TLA	97.29	93.62	
Cat	GRA7	89.7	92.5	[65]
	TLA	84.6	99.3	
Cat	M1 (H4 + H11)	95	100	[72]
	TSA	98	99	
Pig	CM5 (SAG2-GRA1-ROP1L)	93.8	100	[7]
	TLA	100	100	
Horse	CM5 (SAG2-GRA1-ROP1L)	100	100	[7]
	TLA	100	100	
Mink	GRA7	84.2	99.1	[34]
	TSA	68.4	96.4	
Sheep	CM5 (SAG2-GRA1-ROP1L)	100	100	[7]
	TLA	100	100	
Sheep	M14 (GRA1 + SAG2 + ROP1)	100	100	[70]
	TLA	100	100	
Sheep	CM8 (AMA1-SAG2-GRA1-ROP1)	97.92	100	[48]
	TLA	97.92	100	
Goat	CM8 (AMA1-SAG2-GRA1-ROP1)	95.56	100	[48]
	TLA	97.78	100	
Chicken	GRA7	100	98.9	[33]
	TSA	93.8	97.9	
Dog	GRA7	91	97.7	[71]
	TLA	88.1	96.8	

TLA—Whole tachyzoites; TSA—*Toxoplasma gondii* soluble antigens; Se—Sensitivity; Sp—Specificity.

**Table 6:** Comparison of sensitivity, specificity, and level of agreement with a reference test for non-species-specific antibody binding reagents used in enzyme-linked immunoassays to detect antibodies to *Toxoplasma gondii* in various animals.

Conjugate	Species	Positive % (n/N)	Se (%)	Sp (%)	Agreement (Kappa Value)	Reference Test	Reference
Protein A/G	Pig	28.5 (4/14)–76.9 (10/12)	88.6	93.9	0.8	Commercial ELISA	[43]
			88.6	93.9	0.8	MAT	[43]
			84.8	96.8	0.8	WB	[43]
	Cat	100 (11/11)	ND	ND	1	MAT	[43]
	Mice	0 (0/3)–100% (3/3)	ND	ND	1	MAT	[43]
	Seal	0 (0/4)–(14/14)	ND	ND	0.8	MAT	[43]
	Mink	13 (17/131)–15.3(20/131)	68.4–84.2	95.5–99.1	0.7–0.8	WB	[34]
	White-tailed deer	42.2 (113/268)	92	89	ND	ND	[52]
	Alpaca Sheep Goat Horse Dog	57.1 (8/14) Alpaca 58.8 (10/17) Sheep 64.7 (11/17) Goat 13.3 (2/15) Horse 48.2 (27/56) Dog	92 (Overall value)	89 (Overall value)	0.81 (Overall value)	IHA	[68]
	Pig	Not clear	ND	ND	0.9	MAT	[58]
		Not clear	ND	ND	0.8	Commercial ELISA	[58]
	Dog	45.8 (11/24)	ND	ND	0.9	MAT	[58]
	Cat	38.5 (5/13)	ND	ND	0.9	MAT	[58]
Protein A	Goat	22 (132/600)	89.5	97.9	ND	MAT	[55]
	Dog	ND	93	82	0.8	IHA	[69]
	Cat	ND	100	100	1	IHA	[69]
	Dog	84 (178/212)	75-80	80-85	ND	WB	[74]
	Dog	34.7 (42/121)	ND	ND	0.6	MAT	[75]
	Cat	35.5 (16/45)	ND	ND	0.5	MAT	[75]
Protein G	Goat	ND	97	100	0.9	IHA	[69]
	Horse	ND	72	100	0.7	IHA	[69]
	Alpaca	ND	76	95	0.7	IHA	[69]
	Sheep	ND	91	100	0.9	IHA	[69]

n—number positive; N—number tested; Se—Sensitivity; Sp—Specificity; MAT—modified agglutination test; IFAT—indirect fluorescent antibody test; WB—Western blot; IHA—Indirect hemagglutination assay; ND—no data

**Table 7:** Studies testing or describing possible cross-reactivity in detecting *Toxoplasma gondii*-specific antibodies using enzyme-linked immunoassays in various animal species.

Possible Cross-Reactive Species	Host Species	Antigen(s) Used	Comments	Reference
*Neospora caninum*	Pig, Cat, Mice, Seal	Soluble tachyzoite	No cross-reactions were reported	[43]
	Chicken	Sonicated tachyzoite antigens	No cross-reactions were reported Dual infection with *T. gondii* and *N. caninum* was reported	[64]
	Sheep	M14 (GRA1 + SAG2 + ROP1)	No cross-reactions were reported	[70]
	Goat, Sheep	CM6 (AMA1N-SAG2-GRA1-ROP1) CM7 (AMA1C-SAG2-GRA1-ROP1) CM8 (AMA1-SAG2-GRA1-ROP1) CM9 (SAG2-GRA1-ROP1-GRA2)	No cross-reactions were reported	[48]
	Dog	Native purified SAG1 (From tachyzoites)	No cross-reactions were reported	[76]
	Turkey	M12 (GRA7 & GRA8)	No cross-reactions were reported with *N. caninum* positive turkeys Cross-reactions were observed in turkeys which were experimentally infected with *Hammondia hammondi* and turkey- specific *Eimeria* spp.	[62]
	White-tailed deer	Crude extract antigen	No cross-reactions were reported Dual infection with *T. gondii* and *N. caninum* was reported	[51]
*Sarcocystis* spp.	Cattle	Sonicated *T. gondii* antigens	Authors mentioned that some seropositive results for *T. gondii* in cattle could be due to cross-reactions with anti *Neospora* or anti *Sarcocystis* antibodies	[60]
	Pig	Crude rhoptries	No cross-reactions were reported	[77]
*Besnoitia* spp	Pig, Cat Mice, Seal	Soluble tachyzoite	No cross-reactions were reported	[43]
*lsospora suis*	Pig	Tachyzoite lysate	No cross-reactions were reported	[29]
*Trichinella* spp.	Pig, Cat Mice, Seal	Soluble tachyzoite	No cross-reactions were reported	[43]
	Pig	Tachyzoite lysate	No cross-reactions were reported	[29]
*Ascaris suum*	Pig	Tachyzoite lysate	No cross-reactions were reported	[29]
Bacteria species *Salmonella* *Yersinia* *Actinobacillus*	Pig	Tachyzoite lysate	No cross-reactions were reported	[29]
No specific species	Pigs	Purified native SAG1	Authors mentioned the possibility of having low cross-reactions with purified native SAG 1	[49]

## Data Availability

Only published data were included in this systematic review and their citations are provided in the reference list.
